# Treatment Engagement and Outcomes of Mindfulness-Based Cognitive Therapy for Veterans with Psychiatric Disorders

**DOI:** 10.1089/acm.2018.0511

**Published:** 2019-09-12

**Authors:** William R. Marchand, Brandon Yabko, Tracy Herrmann, Heather Curtis, Ryan Lackner

**Affiliations:** ^1^ Whole Health Service, VA Salt Lake City Health Care System, Salt Lake City, UT.; ^2^ Department of Psychiatry, University of Utah School of Medicine, Salt Lake City, UT.

**Keywords:** mindfulness, veterans, meditation, mindfulness-based cognitive therapy, outcomes

## Abstract

***Objectives:*** The aim of this study was to evaluate utilization and outcomes of mindfulness-based cognitive therapy (MBCT) provided to veterans with psychiatric disorders.

***Design:*** Retrospective chart review.

***Settings:*** Veterans Administration Medical Center (VAMC).

***Subjects:*** Ninety-eight veterans with psychiatric illness who were enrolled in an MBCT class between May of 2012 and January of 2016. Subjects were predominately white (95%), male (81%), and >50 years old (74%). The most common psychiatric conditions were any mood disorder (82%) and post-traumatic stress disorder (54%).

***Intervention:*** Eight-week MBCT class.

***Outcome measures:*** Session attendance and pre- to postintervention changes in numbers of emergency department (ED) visits and psychiatric hospitalizations.

***Results:*** The average number of sessions attended was 4.87 of 8 and only 16% were present for all sessions. Veteran demographic variables did not predict the number of MBCT sessions attended. However, both greater numbers of pre-MBCT ED visits (*p* = 0.004) and psychiatric admissions (*p* = 0.031) were associated with attending fewer sessions. Among patients who experienced at least one pre- or post-treatment psychiatric admission in the 2 years pre- or postintervention (*N* = 26, 27%), there was a significant reduction in psychiatric admissions from pre to post (*p* = 0.002). There was no significant change in ED visits (*p* = 0.535).

***Conclusions:*** MBCT may be challenging to implement for veterans with psychiatric illness in, at least some, outpatient VAMC settings due to a high attrition rate. Possible mediation approaches include development of methods to screen for high dropout risk and/or development of shorter mindfulness-based interventions (MBIs) and/or coupling MBIs with pleasurable activities. The finding of a significant decrease in psychiatric hospitalizations from pre- to post-MBCT suggests that prospective studies are warranted utilizing MBCT for veterans at high risk for psychiatric hospitalization.

## Introduction

Mindfulness-Based Cognitive Therapy^1^ is an evidence-based mindfulness-based intervention (MBI) that has been well studied.^2–24^ There is increasing interest in using MBIs for U.S. military members and veterans,^25–28^ and the literature examining the effects of MBIs among veterans is developing.^29–49^ However, almost all studies of mindfulness-based cognitive therapy (MBCT) have been among non-Veteran populations. Many of these studies suggest benefit for prevention of depressive relapse,^2–9^ acute depression,^10,11^ treatment-resistant depression,^12,13^ dysthymia,^14^ bipolar^15–18^ and anxiety^19–21^ spectrum disorders, and adult attention-deficit/hyperactivity disorder,^22^ as well as suicidal ideation.^23,24^ Thus, MBCT could be a useful complementary intervention for veterans with psychiatric conditions. However, the literature regarding use of MBCT among Veteran populations is very limited. One pilot study^40^ suggests that MBCT may be an effective adjunctive therapy for combat-related posttraumatic stress disorder (PTSD) among U.S. military veterans, and another study^50^ among Iranian military veterans has also demonstrated benefit for this condition. In addition, currently, there is an ongoing trial of an adapted version of MBCT for suicidal ideation^26^ among veterans. Given the dearth of literature, studies of MBCT among Veteran populations are needed to determine whether this intervention could be beneficial for those veterans suffering from psychiatric disorders.

The retrospective study reported herein aimed to take an initial step toward understanding the potential of MBCT as an intervention for the Veteran population by reviewing the medical records of veterans with psychiatric illness enrolled in an MBCT class for clinical purposes over a 4-year period. The overarching study aims were to evaluate both treatment engagement and outcomes.

The outcome metrics were pre- versus postintervention numbers of psychiatric hospitalizations and emergency department (ED) visits. These metrics were chosen as both are associated with more severe illness^51,52^ and psychiatric hospitalization has been associated with greater age-adjusted mortality^53^ and suicide risk.^54^ Furthermore, the number of psychiatric hospitalizations has been used in other outcome studies^55,56^ of mental health conditions among veterans. Thus, these metrics are consistent with those used by other investigators and are markers of illness severity.

Additionally, little is known about interventions that may reduce the frequency of psychiatric hospitalizations and ED visits among the Veteran population. The literature suggests^57^ that ambulatory treatment strategies, including aggressive treatment of prodromal symptoms, family crisis therapy, home care, and day hospitalization, may reduce the frequency of hospitalization among community populations, and one study^58^ reported that case management may reduce numbers of hospitalizations among veterans. There is minimal evidence specifically regarding whether MBIs or other evidence-based psychotherapies reduce psychiatric hospitalizations or ED visits in community or Veteran populations. One study^59^ of a community sample reported that multifamily group therapy reduced hospitalizations among individuals with bipolar disorder, and another investigation^60^ reported that short-term dynamic psychotherapy reduced rates of ED visits for medically unexplained symptoms. Regarding MBIs, associations have been reported between MBCT and reduced nonmental health ED visits,^61^ between mindfulness-based stress reduction and decreased ED visits and hospitalizations for medical and psychiatric reasons,^62^ and between a novel MBI and decreased psychiatric hospitalizations among individuals with schizophrenia^63^ in community samples. Thus, while there is some preliminary evidence that dynamic psychotherapy and/or MBIs might impact ED visits and/or hospitalizations in community samples, there is no evidence regarding these interventions among Veteran populations with only one study addressing the topic at all. Thus, in addition to evaluating outcomes associated with MBCT, the study reported herein has the potential to enhance the state of knowledge regarding evidence-based psychotherapies and health care utilization among veterans.

Specific aims were to (1) evaluate treatment engagement using the metric of number of sessions attended; (2) determine if any patient variables would predict treatment engagement; (3) evaluate outcomes; (4) assess whether these outcomes were related to the number of sessions attended; and (5) increase the state of knowledge regarding evidence-based psychotherapies and health care utilization among veterans.

## Materials and Methods

A search of electronic medical records at a large Veterans Administration Medical Center (VAMC) was conducted to identify records of veterans with psychiatric illness who were enrolled in an MBCT class with starting dates between May 2012 and January 2016. Records obtained from the initial search were examined to determine whether each indicated that the Veteran had participated in at least one MBCT class and had at least one psychiatric diagnosis. Records that did not meet these criteria were not included in the subsequent review. Records that met the above criteria were further reviewed and information was extracted for this study.

Eleven separate 8-week MBCT interventions were provided during the period described above. These were provided in 2-h sessions per week over eight consecutive weeks at the Veterans Health Care Administration Salt Lake City Health Care System in the Mental Health Service outpatient clinic. The primary providers were a psychologist and a psychiatrist both of whom have received formal MBCT teacher training and have an extensive formal mindfulness practice. MBCT is a manual-based intervention and was implemented consistent with this previously described protocol^1^ in terms of number of sessions, session agendas, and mindfulness practices taught. Participants were referred for MBCT by their primary mental health provider in response to an e-mail announcement. The only exclusionary criteria for referrals were cognitive impairment, psychosis, and substance abuse with a severity that would interfere with the practice of meditation. Exclusions were not based upon veterans having a diagnosis, but rather the level of severity at the time of the phone screening. The MBCT providers contacted referred patients by phone, conducted a brief screening for current severity of illness, and explained the intervention and answered questions. Veterans who desired to be enrolled were then scheduled into the next available class.

Data extracted from the medical records included demographic information (age, gender, ethnicity, and religious preference) and number of MBCT sessions attended, as well as medical and psychiatric diagnoses. Additionally, the number of ED visits and number of psychiatric admissions were obtained from medical records for the 2 years immediately pre- and postintervention. ED visits were tallied separately from psychiatric admissions such that the total number of visits includes those that resulted in a psychiatric admission as well as those that did not.

The sample consisted of 98 veterans who met review criteria. The cohort was predominately white (93, 95%), male (79, 81%), and >50 years old (72, 74%). Additionally, 79 (81%) had one or more disabilities related to military service. See Table 1 for additional demographic data. All subjects had a least one psychiatric disorder, the most common being any mood disorder (80, 82%), followed by post-traumatic stress disorder (53, 54%). Furthermore, 37 (38%) had a substance use disorder and most (89, 91%) had at least one medical condition. Table 2 outlines the most common psychiatric, substance use disorder, and medical diagnoses. Twenty-six (27%) patients had experienced at least one pre- or post-treatment psychiatric admission in the 2 years pre- or postintervention. Of these, over one-half (57%) were for suicide-related reasons, while the second most common reason (16%) for admission was detoxification related to substance use disorders.

**Table 1.** Demographic Characteristics of Study Population (*n* = 98)

	n *(%)*
Gender	
Male	79 (81)
Female	18 (18)
Transgender	1 (1)
Age, years	
21–29	3 (3)
30–39	15 (15)
40–49	8 (8)
50–59	27 (28)
≥60	45 (46)
Race/Ethnicity	
American Indian or Alaskan Native	1 (1)
Asian or Pacific Islander	1 (1)
Black or African American	2 (2)
Hispanic	2 (2)
White or Caucasian	93 (95)
Religion	
Protestant	9 (9)
Jewish	1 (1)
Latter-day Saints	40 (41)
Roman Catholic	19 (20)
Seventh-day Adventist	2 (2)
Paganism	1 (1)
No preference or unknown	26 (27)

**Table 2.** Diagnoses of Subjects (*n* = 98)

	n *(%)*
Psychiatric diagnoses	
Any psychiatric disorder	98 (100)
Any mood disorder	80 (82)
Depressive spectrum disorder	70 (71)
Bipolar spectrum disorder	10 (10)
Post-traumatic stress disorder	53 (54)
Military sexual trauma	12 (12)
Any anxiety disorder	44 (45)
Attention-deficit/hyperactivity disorder	7 (7)
Other psychiatric diagnoses	6 (6)
Substance use disorder diagnoses	
Any substance use disorder	37 (38)
Alcohol use disorder	15 (15)
Opioid use disorder	2 (2)
Tobacco use disorder	14 (14)
Other substance use disorder	11 (11)
Medical diagnoses	
Any medical diagnosis	89 (91)
Chronic pain	50 (51)
Diabetes	13 (13)
Obesity	43 (44)
Sleep disorder	34 (35)
History of traumatic brain injury	5 (5)
Cardiac disease	15 (15)
Vitamin D deficiency	7 (7)
Hypertension	38 (39)
Hyperlipidemia	32 (33)

Data analyses were conducted to (1) evaluate treatment engagement using the metric of number of sessions attended; (2) determine if any patient variables would predict treatment engagement; (3) evaluate outcomes; and (4) assess whether outcomes were related to the number of sessions attended.

To examine the influence of demographic variables on the number of sessions attended, one-way ANOVA (analysis of variance) tests were utilized to evaluate the impact of age and religious preference (each separated into categories as listed in Table 1), while independent samples *t*-tests were used to assess any impact of gender and presence of a service-connected disability on the number of sessions attended. Linear multiple regression analyses were used to determine whether the presence of a specific psychological or medical diagnosis predicted the number of sessions attended. Only diagnoses that comprised at least 10% of the sample were included in the analysis. Last, linear multiple regression analyses were also conducted to test whether pretreatment psychiatric or ED visits were associated with session attendance.

To evaluate data in terms of completers versus noncompleters, the numeric values of number of sessions attended were first recoded into a dichotomous variable (completer ≥4 and noncompleter <4 sessions attended), and then independent samples *t*-tests and Pearson's chi-square test of independence were used to assess predictors of being a completer as well as differences in outcomes based upon completer status.

Paired *t*-tests were completed to determine if there were significant pre- to postintervention changes in ED visits or psychiatric admissions. Finally, to determine whether the total number of sessions attended predicated outcomes, linear regression analyses were conducted.

This study was approved by the University of Utah Institutional Review Board and the Veterans Administration Salt Lake City Health Care System Research and Development Committee.

## Results

**FIG. 1.**
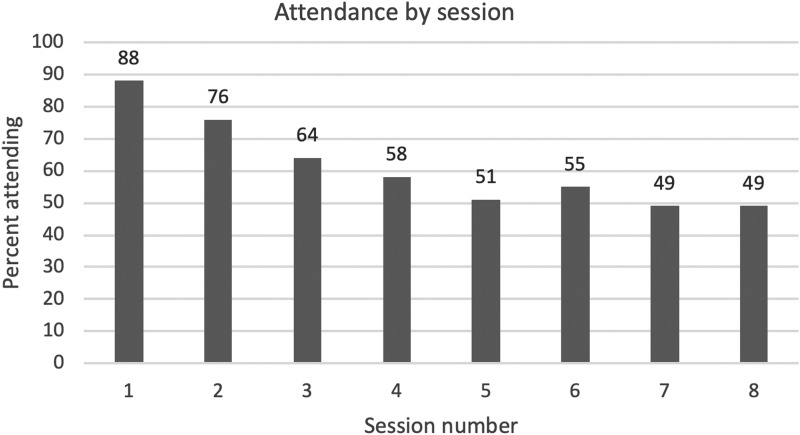
Attendance by session.

The first aim of the study was to assess Veteran treatment engagement for the eight sessions of MBCT. Examination of the number of veterans who attended each session revealed that the average number of sessions attended was 4.87 (SD = 2.4) with a range of 1–8. Figure 1 illustrates that there was a pattern of greater attendance at sessions 1 through 4 compared with sessions 5 through 8. Table 3 indicates the number of sessions actually attended by the number of possible sessions. Only 16 veterans (16%) attended all eight sessions and 3 (3%) attended only one session. Using the definition of completers as attending ≥4 sessions, there were 66 (67%) completers.

**Table 3.** Total Number of Mindfulness-Based Cognitive Therapy Sessions Attended

Number of possible sessions	1	2	3	4	5	6	7	8
Number of veterans attending	3	11	9	10	15	5	19	16
Percent of cohort (*n* = 98), *n* (%)	3	11	9	10	15	5	19	16

Table interpretation: Top row indicates the number of possible sessions that could have been attended (1–8). Middle row indicates the number of veterans who actually attended each possible number of sessions. Third row indicates the percentage of total sample (*n* = 98) who attended each possible number of sessions. For example, column 2 (from the left) indicates that three veterans, which is 3% of the sample, attended only one session. Column 9 indicates that 16 veterans (16% of the sample) attended all 8 sessions.

A second study aim was to determine whether any patient variables predicted the number of sessions attended. Age (*p* = 0.186), religious preference (*p* = 0.500), gender (*p* = 0.326), and service connection (*p* = 0.426) did not predict the number of sessions attended (Table 4). These results remained consistent even after recoding the number of sessions attended into a dichotomous variable of completers versus noncompleters. Furthermore, the presence of a particular psychological or medical diagnosis was not associated with session attendance when separate models were used to predict attendance (Table 5). In contrast, the number of pretreatment ED visits predicted the number of sessions completed such that for every ED visit, session attendance decreased by 0.211 (*p* = 0.004; Cohen's *f*2 = 0.09), approaching a medium effect size by convention (Cohen's *f*2 of 0.02, 0.15, and 0.35 indicate a small, medium, and large effect size, respectively).^64^ Additionally, the number of psychiatric admissions predicted the number of sessions completed such that for every psychiatric admission, session attendance decreased by 0.358 (*p* = 0.031; Cohen's *f*2 = 0.05), a small effect size (Table 6). Finally, none of the patient variables were associated with being a completer versus noncompleter.

**Table 4.** Influence of Demographic Variables on Session Attendance

*Variable*	*ANOVA statistics*			t*-Test statistics*		
	F	*df*	p	t	*df*	p
Age	1.58	4, 93	0.186	—	—	—
Religion	0.925	8, 89	0.500	—	—	—
Gender	—	—	—	−0.99	95	0.326
Service connection	—	—	—	0.80	96	0.426

ANOVA, analysis of variance.

**Table 5.** Influence of a Psychological or Medical Diagnosis on Session Attendance

*Diagnosis*	*Regression statistics*			
	F	*df*	*β*	p
PTSD	2.62	1, 97	−0.779	0.109
Mood disorder	2.49	1, 97	−0.979	0.118
Depressive spectrum disorder	0.39	1, 97	−0.336	0.534
Bipolar disorder	1.14	1, 97	−0.855	0.288
Substance use disorder	0.49	1, 97	−0.351	0.485
Tobacco use disorder	0.22	1, 97	−0.845	0.224
Alcohol use disorder	0.12	1, 97	0.235	0.728
Military sexual trauma	1.47	1, 97	−0.893	0.228
Anxiety disorder	0.12	1, 97	−0.172	0.726
Chronic pain	1.57	1, 97	−0.612	0.215
Diabetes	3.43	1, 97	1.306	0.067
Obesity	0.55	1, 97	0.361	0.463
Sleep disorder	0.75	1, 97	−0.444	0.390
Cardiac disease	0.22	1, 97	0.314	0.643
Hypertension	0.75	1, 97	0.432	0.388
Hyperlipidemia	1.42	1, 97	0.615	0.236

PTSD, posttraumatic stress disorder.

**Table 6.** Association of Pretreatment Psychiatric Admission or Emergency Department Admits with Session Attendance

*Pre-MBCT predictor*	*Regression statistics*				R^*2*^	*Effect size (f^2^)*
	F	*df*	β	p		
ED visits	8.57	1, 97	−0.211	0.004	0.082	0.09
Psychiatric admissions	4.80	1, 97	−0.358	0.031	0.048	0.05

ED, emergency department; MBCT, mindfulness-based cognitive therapy.

A third aim was to determine whether the intervention was associated with any changes in two outcome variables, ED visits and psychiatric admissions. As indicated in Table 7, among patients who experienced at least one pre- or post-treatment psychiatric admission in the 2 years pre- or postintervention (*N* = 26, 27%), there was a reduction in psychiatric admissions from pre to post. The mean numbers of admissions were 2.04 pre- and 0.5 postintervention (*p* = 0.002; Cohen's *d* = 0.69), approaching a large effect size by convention (Cohen's *d* of 0.2, 0.5, and 0.8 indicate a small, medium, and large effect size, respectively).^64^ There was no significant change in ED visits (*p* = 0.535).

**Table 7.** Comparison of Pre- and Postmindfulness-Based Cognitive Therapy Psychiatric Admissions and Emergency Department Visits

*Variable*	*Pretreatment*		*Post-treatment*		*Paired* t*-test statistics*			
	M	*SD*	M	*SD*	t	*df*	p	*Effect size (d)*
Psychiatric admissions	2.04	2.25	0.50	0.71	3.54	25	0.002	0.69
ED visits	2.96	3.59	3.41	2.63	−0.62	68	0.535	0.07

The final aim was to determine if the number of sessions attended impacted outcomes. There was no significant association between the total number of sessions attended and outcomes (Table 8), nor with completer versus noncompleter status.

**Table 8.** Association Between Number of Sessions Completed and Clinical Outcomes

*Clinical outcome*	*Regression statistics*			
	F	*df*	*β*	p
Number of post-MBCT				
Psychiatric admissions	2.39	1, 97	−0.027	0.126
Number of post-MBCT				
ED visits	2.18	1, 97	−0.164	0.143

## Discussion

To the authors' knowledge, this is the first article to report on utilization and outcomes from MBCT delivered for clinical purposes in a VAMC outpatient setting and one of the few articles to report on any aspect of utilizing this intervention among a Veteran population.

The first key finding is that the attrition rate was high. For all classes, the average number of sessions attended was 4.87 and only 16 veterans (16%) attended all sessions. Figure 1 indicates attendance by session for all 11 cohorts and illustrates consistent decreased attendance across sessions 1 through 4 and then stabilization with ∼50% attendance of sessions 5 through 8. Using the definition of completers as attending ≥4 sessions, there were 66 (67%) completers.

There is limited literature available to compare the completer rates that the authors report with that of other similar investigations. However, King et al.^40^ conducted a pilot study of MBCT for veterans with PTSD and found a 75% completer rate. Other studies of MBIs among veterans completed by Kearney et al. found that for a 12-session loving-kindness intervention,^65^ 74% of participants attended 9–12 sessions. Other studies of MBSR among veterans by this group reported completer rates of 73%,^37^ 74%,^35^ and 84%.^36^ Rates of completion for behavioral medicine trials for nonveterans have been reported as ranging from 41% to 90%^66^ and 50% to 63% for psychopharmacology^67^ trials and 63% to 100% for other randomized controlled trials.^68^ Thus, the completer rate reported herein is somewhat lower than that reported by some other investigators. This might be explained, in part, by the fact that this was a relatively ill population; 81% had one or more disabilities related to military service, 82% had a mood disorder, 54% had post-traumatic stress disorder (53, 54%), and finally, 91% had at least one medical condition.

If confirmed by additional studies, these results suggest that MBCT engagement may be challenging for veterans, but this is likely due, at least in part, to outpatient engagement difficulties more broadly. It is not possible to conclude that this barrier is specific to MBCT because, as described above, this challenge exists more generally for outpatient treatment among both Veteran^35–37,65^ and community samples.^66–68^ One approach to mediate MBCT attrition could be to develop MBIs that are of shorter duration. Figure 1 suggests that three to four sessions might be optimal for this population, and some evidence^29,31,45,69^ suggests that shorter MBIs can be effective for veterans. However, this approach could negatively impact the effect size of the intervention. Other programmatic modifications that could be considered include offering orientation groups, peer support, intensive follow-up and engagement before discharge for hospitalized patients, and follow-up phone calls after each MBCT session, as well as targeting specific populations and/or problems based on gender, era of service, and/or diagnoses.

Another approach could be development of screening methods to identify veterans at risk of attrition. Thus, analyses were conducted to determine whether any Veteran characteristics would serve as predictors of dropout in this sample. Results indicated that gender, age, religious preference, and presence of a service-connected disability, as well as the presence of any specific psychiatric or medical diagnosis, did not predict the total number of MBCT sessions attended (Tables 4 and 5).

The second key finding of this study was that both the numbers of preintervention ED visits and psychiatric admissions were significantly associated with the number of sessions attended (Table 6). For every ED visit, attendance decreased by 0.211, and for every psychiatric admission, attendance decreased by 0.358. A study by Crane and Williams^70^ on MBCT attrition in a community population found that those who dropped out of MBCT were significantly younger than those who completed treatment, less likely to be on antidepressants, had higher levels of depressive rumination and brooding, and showed significantly greater levels of problem-solving deterioration following mood challenge. Although there are some discrepancies, taken together, the authors' findings and those of Crane and Williams suggest that MBCT attrition may be, at least partly, associated with individual symptom expression characteristics and/or severity or instability of illness, as indicated by utilization of ED and psychiatric inpatient facilities. It is possible that the higher levels of depressive rumination and problem-solving deterioration found by Crane and Williams could be mediating factors of greater utilization of EDs and psychiatric hospitalizations. However, other unidentified variables that are associated with utilization of EDs and/or inpatient psychiatric facilities could play a role, such as psychosocial issues, homelessness, and/or transportation challenges. Additional studies should be aimed at further disambiguating predictors of attrition as well as whether predictors might be utilized to develop criteria to assess whether veterans are appropriate for an 8-week MBCT.

The final key finding reported herein is that in this population, MBCT participation was associated with significant pre- to postintervention reductions in the numbers of psychiatric hospitalizations (Table 7). Among the 26 veterans who experienced a psychiatric admission within the 2 years pre- or postintervention, the mean number of admissions dropped significantly from 2.04 to 0.5 pre to post. This finding does not prove cause and effect; nonetheless, it is an intriguing result and suggests the need for prospective studies among veterans who are high utilizers of psychiatric hospitalization. If confirmed by more rigorous studies, MBCT may be an effective intervention for veterans with psychiatric illness who have a high risk of hospitalization. Interestingly, neither the number of sessions attended nor completer versus noncompleter status predicted the reduction in psychiatric admissions (Table 8). If confirmed, this result could support development of shorter duration MBIs for this population, as suggested above.

There are several limitations that must be taken into consideration when interpreting the results of this study. This study was retrospective rather than a randomized controlled trial. Selection bias is a concern as veterans were selected for referral by their primary treating mental health provider for various reasons. In addition, the most common psychiatric diagnoses were depressive spectrum, PTSD, or other anxiety spectrum disorders and the population was predominately older white males. Thus, results may not be generalizable to other Veteran or community populations or other psychiatric disorders. In addition, while associations are reported herein, it is important to note that this work did not demonstrate cause and effect. There may be other mediating factors influencing both predictive and outcome variables, and prospective studies will be needed to confirm the findings reported in this article.

## Conclusions

MBCT and other MBIs are being utilized for veterans with psychiatric illness, although there is very limited literature supporting the use of MBCT for this population. Despite some methodological limitations, findings reported herein provide the groundwork for future studies that will be necessary to understand whether and how to best utilize MBCT for this population.

MBCT may be challenging to implement for veterans with psychiatric illness in, at least some, outpatient VAMC settings due to a high attrition rate. If replicated, these results suggest that processes will be needed to mediate attrition for MBCT and perhaps other 8-week MBIs.

The significant decrease in psychiatric hospitalizations from pre- to post-MBCT does not demonstrate cause and effect; however, it suggests that prospective studies are warranted, utilizing MBCT for veterans at high risk for psychiatric hospitalization.

## References

[1] Segal ZV, Willliams JMG, Teasdale JD Mindfulness-Based Cognitive Therapy for Depression, 2nd ed. New York: The Guilford Press, 2012.

[2] Godfrin KA, van Heeringen C The effects of mindfulness-based cognitive therapy on recurrence of depressive episodes, mental health and quality of life: A randomized controlled study. Behav Res Ther 2010;48:738–746 20462570 10.1016/j.brat.2010.04.006

[3] Segal ZV, Bieling P, Young T, et al. Antidepressant monotherapy vs sequential pharmacotherapy and mindfulness-based cognitive therapy, or placebo, for relapse prophylaxis in recurrent depression. Arch Gen Psychiatry 2010;67:1256–1264 21135325 10.1001/archgenpsychiatry.2010.168 PMC3311113

[4] Fjorback LO, Arendt M, Ornbol E, et al. Mindfulness-based stress reduction and mindfulness-based cognitive therapy: A systematic review of randomized controlled trials. Acta Psychiatr Scand 2011:124:102–119 21534932 10.1111/j.1600-0447.2011.01704.x

[5] Piet J, Hougaard E The effect of mindfulness-based cognitive therapy for prevention of relapse in recurrent major depressive disorder: A systematic review and meta-analysis. Clin Psychol Rev 2011;31:1032–1040 21802618 10.1016/j.cpr.2011.05.002

[6] Williams JM, Crane C, Barnhofer T, et al. Mindfulness-based cognitive therapy for preventing relapse in recurrent depression: A randomized dismantling trial. J Consult Clin Psychol 2014;82:275–286 24294837 10.1037/a0035036 PMC3964149

[7] Kuyken W, Warren FC, Taylor RS, et al. Efficacy of mindfulness-based cognitive therapy in prevention of depressive relapse: An individual patient data meta-analysis from randomized trials. JAMA Psychiatry 2016;73:565–574 27119968 10.1001/jamapsychiatry.2016.0076 PMC6640038

[8] Segal ZV, Walsh KM Mindfulness-based cognitive therapy for residual depressive symptoms and relapse prophylaxis. Curr Opin Psychiatry 2016;29:7–12 26575299 10.1097/YCO.0000000000000216 PMC4706736

[9] Cladder-Micus MB, Speckens AEM, Vrijsen JN, et al. Mindfulness-based cognitive therapy for patients with chronic, treatment-resistant depression: A pragmatic randomized controlled trial. Depress Anxiety 2018;35:914–924 30088834 10.1002/da.22788 PMC6175087

[10] Manicavasgar V, Parker G, Perich T Mindfulness-based cognitive therapy vs cognitive behaviour therapy as a treatment for non-melancholic depression. J Affect Disord 2011;130:138–144 21093925 10.1016/j.jad.2010.09.027

[11] van Aalderen JR, Donders AR, Giommi F, et al. The efficacy of mindfulness-based cognitive therapy in recurrent depressed patients with and without a current depressive episode: A randomized controlled trial. Psychol Med 2012;42:989–1001 22017808 10.1017/S0033291711002054

[12] Chiesa A, Castagner V, Andrisano C, et al. Mindfulness-based cognitive therapy vs. psycho-education for patients with major depression who did not achieve remission following antidepressant treatment. Psychiatry Res 2015;226:474–483 25744325 10.1016/j.psychres.2015.02.003

[13] Eisendrath SJ, Gillung E, Delucchi KL, et al. A randomized controlled trial of mindfulness-based cognitive therapy for treatment-resistant depression. Psychother Psychosom 2016;85:99–110 26808973 10.1159/000442260 PMC4756643

[14] Hamidian S, Omidi A, Mousavinasab SM, Naziri G Comparison of the effect of mindfulness-based cognitive therapy accompanied by pharmacotherapy with pharmacotherapy alone in treating dysthymic patients. Iran Red Crescent Med J 2013;15:239–244 23984005 10.5812/ircmj.8024 PMC3745754

[15] Williams JM, Alatiq Y, Crane C, et al. Mindfulness-based cognitive therapy (MBCT) in bipolar disorder: Preliminary evaluation of immediate effects on between-episode functioning. J Affect Disord 2008;107:275–279 17884176 10.1016/j.jad.2007.08.022 PMC2881943

[16] Weber B, Jermann F, Gex-Fabry M, et al. Mindfulness-based cognitive therapy for bipolar disorder: A feasibility trial. Eur Psychiatry 2010;25:334–337 20561769 10.1016/j.eurpsy.2010.03.007

[17] Deckersbach T, Holzel BK, Eisner LR, et al. Mindfulness-based cognitive therapy for nonremitted patients with bipolar disorder. CNS Neurosci Ther 2012;18:133–141 22070469 10.1111/j.1755-5949.2011.00236.x PMC3738005

[18] Lovas DA, Schuman-Olivier Z Mindfulness-based cognitive therapy for bipolar disorder: A systematic review. J Affect Disord 2018;240:247–261 30086469 10.1016/j.jad.2018.06.017 PMC7448295

[19] Kim B, Lee SH, Kim YW, et al. Effectiveness of a mindfulness-based cognitive therapy program as an adjunct to pharmacotherapy in patients with panic disorder. J Anxiety Disord 2010;24:590–595 20427148 10.1016/j.janxdis.2010.03.019

[20] Lovas DA, Barsky AJ Mindfulness-based cognitive therapy for hypochondriasis, or severe health anxiety: A pilot study. J Anxiety Disord 2010;24:931–935 20650601 10.1016/j.janxdis.2010.06.019

[21] Piet J, Hougaard E, Hecksher MS, Rosenberg NK A randomized pilot study of mindfulness-based cognitive therapy and group cognitive-behavioral therapy for young adults with social phobia. Scand J Psychol 2010;51:403–410 20210911 10.1111/j.1467-9450.2009.00801.x

[22] Janssen L, Kan CC, Carpentier PJ, et al. Mindfulness-based cognitive therapy v. treatment as usual in adults with ADHD: A multicentre, single-blind, randomised controlled trial. Psychol Med 2019;49:55–65 29486807 10.1017/S0033291718000429

[23] Barnhofer T, Crane C, Brennan K, et al. Mindfulness-based cognitive therapy (MBCT) reduces the association between depressive symptoms and suicidal cognitions in patients with a history of suicidal depression. J Consult Clin Psychol 2015;83:1013–1020 26302249 10.1037/ccp0000027 PMC4655869

[24] Forkmann T, Wichers M, Geschwind N, et al. Effects of mindfulness-based cognitive therapy on self-reported suicidal ideation: Results from a randomised controlled trial in patients with residual depressive symptoms. Compr Psychiatry 2014;55:1883–1890 25218397 10.1016/j.comppsych.2014.08.043

[25] Gallegos AM, Cross W, Pigeon WR Mindfulness-based stress reduction for veterans exposed to military sexual trauma: Rationale and implementation considerations. Mil Med 2015;180:684–689 26032384 10.7205/MILMED-D-14-00448

[26] Kline A, Chesin M, Latorre M, et al. Rationale and study design of a trial of mindfulness-based cognitive therapy for preventing suicidal behavior (MBCT-S) in military veterans. Contemp Clin Trials 2016;50:245–252 27592123 10.1016/j.cct.2016.08.015

[27] Kopacz MS, Connery AL, Bishop TM, et al. Moral injury: A new challenge for complementary and alternative medicine. Complement Ther Med 2016;24:29–33 26860798 10.1016/j.ctim.2015.11.003

[28] Stanton MV, Matsuura J, Fairchild JK, et al. Mindfulness as a weight loss treatment for veterans. Front Nutr 2016;3:30 27574603 10.3389/fnut.2016.00030 PMC4983552

[29] Bergen-Cico D, Possemato K, Pigeon W Reductions in cortisol associated with primary care brief mindfulness program for veterans with PTSD. Med Care 2014;52(Suppl 5):S25–S31 10.1097/MLR.0000000000000224 25397819

[30] Bremner JD, Mishra S, Campanella C, et al. A pilot study of the effects of mindfulness-based stress reduction on post-traumatic stress disorder symptoms and brain response to traumatic reminders of combat in operation enduring freedom/operation Iraqi freedom combat veterans with post-traumatic stress disorder. Front Psychiatry 2017;8:157 28890702 10.3389/fpsyt.2017.00157 PMC5574875

[31] Carlson KJ, Silva SG, Langley J, Johnson C Mindful-Veteran: The implementation of a brief stress reduction course. Complement Ther Clin Pract 2013;19:89–96 23561066 10.1016/j.ctcp.2012.12.003

[32] Cole MA, Muir JJ, Gans JJ, et al. Simultaneous treatment of neurocognitive and psychiatric symptoms in veterans with post-traumatic stress disorder and history of mild traumatic brain injury: A pilot study of mindfulness-based stress reduction. Mil Med 2015;180:956–963 26327547 10.7205/MILMED-D-14-00581

[33] Cushing RE, Braun KL Mind-body therapy for military veterans with post-traumatic stress disorder: A systematic review. J Altern Complement Med 2018;24:106–114 28880607 10.1089/acm.2017.0176

[34] Held P, Owens GP, Monroe JR, Chard KM Increased mindfulness skills as predictors of reduced trauma-related guilt in treatment-seeking veterans. J Trauma Stress 2017;30:425–431 28741736 10.1002/jts.22209

[35] Kearney DJ, McDermott K, Malte C, et al. Association of participation in a mindfulness program with measures of PTSD, depression and quality of life in a veteran sample. J Clin Psychol 2012;68:101–116 22125187 10.1002/jclp.20853

[36] Kearney DJ, McDermott K, Malte C, et al. Effects of participation in a mindfulness program for veterans with posttraumatic stress disorder: A randomized controlled pilot study. J Clin Psychol 2013;69:14–27 22930491 10.1002/jclp.21911

[37] Kearney DJ, Simpson TL, Malte CA, et al. Mindfulness-based stress reduction in addition to usual care is associated with improvements in pain, fatigue, and cognitive failures among veterans with Gulf War Illness. Am J Med 2016;129:204–214 26519614 10.1016/j.amjmed.2015.09.015

[38] King AP, Block SR, Sripada RK, et al. Altered default mode network (DMN) resting state functional connectivity following a mindfulness-based exposure therapy for posttraumatic stress disorder (PTSD) in combat veterans of Afghanistan and Iraq. Depress Anxiety 2016;33:289–299 27038410 10.1002/da.22481

[39] King AP, Block SR, Sripada RK, et al. A pilot study of mindfulness-based exposure therapy in OEF/OIF combat veterans with PTSD: Altered medial frontal cortex and amygdala responses in social-emotional processing. Front Psychiatry 2016;7:154 27703434 10.3389/fpsyt.2016.00154 PMC5028840

[40] King AP, Erickson TM, Giardino ND, et al. A pilot study of group mindfulness-based cognitive therapy (MBCT) for combat veterans with posttraumatic stress disorder (PTSD). Depress Anxiety 2013;30:638–645 23596092 10.1002/da.22104 PMC4373594

[41] Kluepfel L, Ward T, Yehuda R, et al. The evaluation of mindfulness-based stress reduction for veterans with mental health conditions. J Holist Nurs 2013;31:248–255; quiz 256–257 23863274 10.1177/0898010113495975

[42] O'Malley PG In veterans with PTSD, mindfulness-based group therapy reduced symptom severity. Ann Intern Med 2015;163:JC9 10.7326/ACPJC-2015-163-12-009 26666811

[43] Omidi A, Mohammadi A, Zargar F, Akbari H Efficacy of mindfulness-based stress reduction on mood States of veterans with post-traumatic stress disorder. Arch Trauma Res 2013;1:151–154 24396769 10.5812/atr.8226 PMC3876494

[44] Polusny MA, Erbes CR, Thuras P, et al. Mindfulness-based stress reduction for posttraumatic stress disorder among veterans: A randomized clinical trial. JAMA 2015;314:456–465 26241597 10.1001/jama.2015.8361

[45] Possemato K, Bergen-Cico D, Treatman S, et al. A randomized clinical trial of primary care brief mindfulness training for veterans with PTSD. J Clin Psychol 2016;72:179–193 26613203 10.1002/jclp.22241

[46] Serpa JG, Taylor SL, Tillisch K Mindfulness-based stress reduction (MBSR) reduces anxiety, depression, and suicidal ideation in veterans. Med Care 2014;52(Suppl 5):S19–S24 25397818 10.1097/MLR.0000000000000202

[47] Stephenson KR, Simpson TL, Martinez ME, Kearney DJ Changes in mindfulness and posttraumatic stress disorder symptoms among veterans enrolled in mindfulness-based stress reduction. J Clin Psychol 2017;73:201–217 27152480 10.1002/jclp.22323

[48] Wahbeh H, Goodrich E, Goy E, Oken BS Mechanistic pathways of mindfulness meditation in combat veterans with posttraumatic stress disorder. J Clin Psychol 2016;72:365–383 26797725 10.1002/jclp.22255 PMC4803530

[49] Dahm KA, Meyer EC, Neff KD, et al. Mindfulness, self-compassion, posttraumatic stress disorder symptoms, and functional disability in U.S. Iraq and Afghanistan War Veterans. J Trauma Stress 2015;28:460–464 26426991 10.1002/jts.22045 PMC5032647

[50] Jasbi M, Sadeghi Bahmani D, Karami G, et al. Influence of adjuvant mindfulness-based cognitive therapy (MBCT) on symptoms of post-traumatic stress disorder (PTSD) in veterans—Results from a randomized control study. Cogn Behav Ther 2018;47:431–446 29893182 10.1080/16506073.2018.1445773

[51] Camacho A, Ng B, Bejarano A, et al. Crisis visits and psychiatric hospitalizations among patients attending a community clinic in rural Southern California. Community Ment Health J 2012;48:133–137 20924788 10.1007/s10597-010-9350-0 PMC3157591

[52] Schmutte T, Dunn C, Sledge W Characteristics of inpatients with a history of recurrent psychiatric hospitalizations: A matched-control study. Psychiatr Serv 2009;60:1683–1685 19952162 10.1176/ps.2009.60.12.1683

[53] Haklai Z, Goldberger N, Stein N, et al. The mortality risk among persons with psychiatric hospitalizations. Isr J Psychiatry Relat Sci 2011;48:230–239 22572086

[54] Deisenhammer EA, Huber M, Kemmler G, et al. Psychiatric hospitalizations during the last 12 months before suicide. Gen Hosp Psychiatry 2007;29:63–65 17189748 10.1016/j.genhosppsych.2006.09.007

[55] Fasoli DR, Glickman ME, Eisen SV Predisposing characteristics, enabling resources and need as predictors of utilization and clinical outcomes for veterans receiving mental health services. Med Care 2010;48:288–295 20355260 10.1097/mlr.0b013e3181cafbe3

[56] Burningham Z, Leng J, Peters CB, et al. Predicting psychiatric hospitalizations among elderly veterans with a history of mental health disease. EGEMS (Wash DC) 2018;6:7 29881765 10.5334/egems.207 PMC5982950

[57] Herz MI Treatment strategies for reducing costs of acute psychiatric hospitalization. Int J Partial Hosp 1985;3:81–90 10277558

[58] Mares A, McGuire J Reducing psychiatric hospitalization among mentally ill veterans living in board-and-care homes. Psychiatr Serv 2000;51:914–921 10875958 10.1176/appi.ps.51.7.914

[59] Solomon DA, Keitner GI, Ryan CE, et al. Preventing recurrence of bipolar I mood episodes and hospitalizations: Family psychotherapy plus pharmacotherapy versus pharmacotherapy alone. Bipolar Disord 2008;10:798–805 19032711 10.1111/j.1399-5618.2008.00624.x

[60] Abbass A, Campbell S, Magee K, Tarzwell R Intensive short-term dynamic psychotherapy to reduce rates of emergency department return visits for patients with medically unexplained symptoms: Preliminary evidence from a pre-post intervention study. CJEM 2009;11:529–534 19922712 10.1017/s1481803500011799

[61] Kurdyak P, Newman A, Segal Z Impact of mindfulness-based cognitive therapy on health care utilization: A population-based controlled comparison. J Psychosom Res 2014;77:85–89 25077847 10.1016/j.jpsychores.2014.06.009

[62] McCubbin T, Dimidjian S, Kempe K, et al. Mindfulness-based stress reduction in an integrated care delivery system: One-year impacts on patient-centered outcomes and health care utilization. Perm J 2014;18:4–9 10.7812/TPP/14-014 PMC4206164 25662520

[63] Chien WT, Lee IY The mindfulness-based psychoeducation program for Chinese patients with schizophrenia. Psychiatr Serv 2013;64:376–379 23412024 10.1176/appi.ps.002092012

[64] Cohen J Statistical Power Analysis or the Behavioral Sciences, 2nd ed. Hillsdale, NJ: Lawrence Erlbaum Associates, 1988

[65] Kearney DJ, Malte CA, McManus C, et al. Loving-kindness meditation for posttraumatic stress disorder: A pilot study. J Trauma Stress 2013;26:426–434 23893519 10.1002/jts.21832

[66] Davis MJ, Addis ME, Predictors of attrition from behavioral medicine treatments. Ann Behav Med 1999;21:339–349 10721442 10.1007/BF02895967

[67] Leon AC, Mallinckrodt CH, Chuang-Stein C, et al. Attrition in randomized controlled clinical trials: Methodological issues in psychopharmacology. Biol Psychiatry 2006;59:1001–1005 16503329 10.1016/j.biopsych.2005.10.020

[68] Hewitt CE, Kumaravel B, Dumville JC, et al. Assessing the impact of attrition in randomized controlled trials. J Clin Epidemiol 2010;63:1264–1270 20573482 10.1016/j.jclinepi.2010.01.010

[69] DiNardo M, Saba S, Greco CM, et al. A mindful approach to diabetes self-management education and support for veterans. Diabetes Educ 2017;43:608–620 29078735 10.1177/0145721717738019

[70] Crane C, Williams JM Factors associated with attrition from mindfulness-based cognitive therapy in patients with a history of suicidal depression. Mindfulness (N Y) 2010;1:10–20 21125023 10.1007/s12671-010-0003-8 PMC2987524

